# Podcasting in Nursing and Midwifery Education and Continuing Professional Development: A Scoping Review

**DOI:** 10.3390/healthcare14182916

**Published:** 2026-09-09

**Authors:** Abdulqadir J. Nashwan, Jibin Kunjavara, Rebecca George, Mahmoud A. Khedr, Yasmine M. Osman, Anas H. Khalifeh, Fadwa Al-halaiqa

**Affiliations:** 1 Nursing and Midwifery Research Department, Hamad Medical Corporation, Doha P.O. Box 3050, Qatar; jkunjavara@hamad.qa; 2 Department of Accredited Training Provider (FCHS-ATP), Fatima College of Health Sciences, Ajman P.O. Box 3798, United Arab Emirates; rebecca.george@actvet.gov.ae; 3 Psychiatric and Mental Health Nursing Department, Faculty of Nursing, Alexandria University, Alexandria 21526, Egypt; mahmoud.khedr@alexu.edu.eg; 4 Department of Obstetrics and Gynecological Nursing, Faculty of Nursing, Zagazig University, Zagazig 44519, Egypt; dry.shady12@gmail.com; 5 Department of Community and Mental Health Nursing, Faculty of Nursing, Zarqa University, P.O. Box 2000, Zarqa 13110, Jordan; akhalifeh@zu.edu.jo; 6 Department of Clinical Affairs, College of Nursing, QU Health, Qatar University, Doha P.O. Box 2713, Qatar; f.alhalaiqa@qu.edu.qa

**Keywords:** nursing, midwifery, nursing education, podcasting, professional education, digital media

## Abstract

**Background**: Digital innovations have transformed health professions education, with podcasting emerging as a flexible, learner-centered educational modality. Podcasts support asynchronous, mobile, and self-directed learning, enabling access to educational content beyond traditional classroom environments. However, evidence regarding their effectiveness, integration strategies, and impact on educational and practice outcomes remains fragmented. **Aim/Objective**: This review aimed to map the existing literature on the use of podcasting in nursing and midwifery education and continuing professional development (CPD), identify how podcasts are used, summarize reported benefits and limitations, and highlight gaps for future research. Design: Scoping review. **Methods**: A scoping review was conducted and reported following the Preferred Reporting Items for Systematic Reviews and Meta-Analyses Extension for Scoping Reviews (PRISMA-ScR) guidelines. Eligibility criteria were developed using the Population–Concept–Context (PCC) framework to define the review scope. A comprehensive literature search was performed across PubMed/MEDLINE, Scopus, Embase, CINAHL, Google Scholar, ProQuest, and OpenGrey to identify studies published in English between January 2005 and December 2024. Two reviewers independently screened titles, abstracts, and full-text articles against the predefined eligibility criteria. Data were extracted using a standardized charting form, and the included studies were synthesized using descriptive statistics and thematic analysis to map the characteristics and educational applications. They reported outcomes of podcasting in nursing and midwifery education. **Results**: Twenty-four studies were included. Podcasting was associated with enhanced learning flexibility, learner engagement, and knowledge retention in both academic and CPD contexts. It supported asynchronous and self-directed learning while reinforcing key concepts. Challenges included variable content quality, limited integration of assessment, and scarce evidence linking podcast use to clinical outcomes. **Conclusions**: Podcasting is a promising adjunct to nursing and midwifery education and CPD. Formal integration into curricula and professional development frameworks is recommended. Further research should focus on longitudinal outcomes, low- and middle-income settings, and impacts on clinical practice and interprofessional learning.

## 1. Introduction

Technological advancements have transformed healthcare education, with digital learning tools central to curriculum development and training [1]. Podcasting, a powerful and flexible educational medium, has gained prominence. The nursing and midwifery education landscape has evolved through the integration of digital tools. Initially for entertainment and journalism, podcasts now serve academic purposes, including healthcare education. Asynchronous, on-demand audio recordings allow learners to engage with content in a self-paced, mobile-friendly, and relevant manner [2].

The use of podcasting in health professions education reflects a shift towards multimodal and microlearning strategies [3]. In nursing and midwifery, it offers a learner-centered approach, enabling access anytime and promoting autonomy, aligning with 21st-century learners’ preferences for convenience, personalization, and control [4]. Students increasingly use mobile devices and digital platforms, making podcasts an ideal supplement to traditional methods [5]. Podcasts enhance traditional classroom learning by reinforcing concepts, supporting exam prep, and encouraging reflection. The literature suggests podcasting boosts engagement, motivation, and learning outcomes across healthcare topics [6].

Podcasting helps nursing and midwifery students overcome challenges such as balancing coursework and clinical placements, limited in-person lecture options, and high cognitive load. It complements classroom learning by reinforcing concepts, aiding exam prep, and encouraging reflection. Educators use podcasts across settings to cover topics such as clinical procedures, ethics, health promotion, and global health. Contemporary health professions education is increasingly shaped by the need for lifelong learning and resilient learning, enabling healthcare professionals to continually update their knowledge and competencies in response to rapidly evolving clinical evidence, technological innovation, and changing healthcare needs [2].

Podcasting, initially popularized in entertainment and journalism, has gained momentum in academic and clinical training [7]. In nursing and midwifery education, it reflects broader shifts toward blended and microlearning formats, especially as higher education seeks to accommodate diverse learners. During emergencies like pandemics or disasters, podcasting offers a resilient educational option [8]. The COVID-19 pandemic further accelerated the use of digital platforms, making podcasts key as both supplemental and primary learning tools. Advances in digital technologies have transformed educational delivery, providing flexible, learner-centered approaches that support continuous professional development across diverse learning environments. At the same time, learners increasingly prefer self-directed, asynchronous, and mobile learning experiences that can be accessed at their convenience and integrated into busy academic and clinical schedules [9,10].

Consequently, digital educational strategies including e-learning, mobile learning, simulation, and podcasting have become important components of contemporary health professions education, offering accessible and flexible opportunities to support knowledge acquisition, learner engagement, and professional development. Podcasting aligns with adult learning theories such as self-directed learning and constructivism, emphasizing autonomy, reflection, and contextual understanding, all of which are crucial in healthcare education. It enables learners to pause, replay, and review content, increasing engagement and retention [11]. Research shows it bridges theory and practice, supports flipped classrooms, and enhances access in under-resourced or dispersed settings [6]. Additionally, podcasting promotes interprofessional and global collaboration, as seen in multi-country nursing student studies [12].

Continuing professional development (CPD) differs from pre-licensure education as it focuses on post-qualification learning, maintaining competence, and practice-based knowledge rather than foundational skills [13]. Educational podcasts are valuable tools in professional development, supporting flexible, self-directed learning and engagement. To maximize their impact, educators and organizations should highlight the adaptability of podcasts, their support for reflective and social learning, and their relevance to practice [14]. Their inclusion in this review reflects podcasting’s role as a flexible learning modality across professional development, not a substitute for pre-licensure education. In CPD, podcasts address barriers such as time, staffing, and geographic limitations [15], offering concise content on clinical guidelines, leadership, patient safety, and policy changes, thereby enabling clinicians to maintain competence without leaving practice [16]. The collaborative, global nature of podcast production, involving clinicians, educators, and patients, promotes diverse perspectives and community in professional learning [17].

Despite podcasts’ popularity, challenges persist in implementation, quality assurance, and evaluation. Pedagogical rigor, content quality, and assessment methods vary across studies. Educational podcasts differ in design, with variations in quality, accuracy, and alignment with learning goals. Some are developed systematically with pedagogical input, while others lack clear frameworks [18]. While student feedback is positive, evidence on long-term learning, behavior change, and clinical application is limited. Many rely on self-report, small samples, or short-term metrics, limiting generalizability. There is no consensus on integrating podcasts into curricula and CPD programs [3].

Podcasting is increasingly used in health professions education, but research on its effectiveness and implementation in nursing, midwifery, and CPD is limited and fragmented. A comprehensive scoping review is needed to map the existing literature, focusing on patterns of use, outcomes, benefits, challenges, and research gaps. This review defines CPD as structured learning to update professional skills, including podcasts aimed at professionals rather than the public, to distinguish between educational and public health initiatives.

## 2. Materials and Methods

### 2.1. Methods

This scoping review followed the methodological approach proposed by Arksey and O’Malley [19] and was reported in accordance with the PRISMA-ScR guidelines. The five stages of the framework, formulating the research questions, identifying relevant studies, selecting eligible sources, charting the data, and collating and synthesizing the results, guided the review process.

### 2.2. Review Questions

In the first stage of the scoping review, clear research questions were developed to define the review’s scope and objectives. These questions aimed to examine the extent of the literature on podcasting in nursing and midwifery education, explore its use in continuing professional development, identify reported outcomes and challenges, and highlight gaps in the existing evidence base.

- What is the current scope of the literature on podcasting in nursing and midwifery education?
- How is podcasting being utilized in CPD for nurses and midwives?
- What are the reported outcomes, benefits, challenges, and limitations of podcasting in nursing and midwifery education and CPD?
- What gaps exist in the literature?

### 2.3. Identifying Relevant Studies

Identifying relevant studies was the second stage of the review process. A comprehensive and systematic search strategy was developed to capture the literature on the use of podcasting in nursing and midwifery education and continuing professional development (CPD). Electronic database searches were conducted in MEDLINE (PubMed), CINAHL, Scopus, and Embase. To ensure comprehensive coverage, the grey literature was also searched using ProQuest Dissertations and Theses, OpenGrey, and Google Scholar.

For ProQuest Dissertations and Theses, a targeted search was undertaken using predefined keywords related to podcasting in nursing and midwifery education, with filters applied to relevant disciplinary fields. OpenGrey was searched using the same keyword strategy, with a focus on educational reports, guidelines, and non-peer-reviewed materials. The Google Scholar search involved screening the first 100 results across five pages using identical search terms. This grey literature search approach was informed by methodological guidance from Miake-Lye [20] which recommends systematic, transparent screening procedures to improve the identification of relevant non-peer-reviewed sources.

### 2.4. Eligibility Criteria

Eligibility was established using the Population–Concept–Context (PCC) framework recommended for scoping reviews. Population: The population included undergraduate and postgraduate nursing students, midwifery students, registered nurses, registered midwives, nurse educators, clinical educators, preceptors, and other nursing or midwifery professionals participating in educational or continuing professional development (CPD) activities. Concept: The concept of interest was the use of podcasting as an educational intervention or learning resource, including audio or video podcasts used for teaching, learning, curriculum delivery, knowledge translation, skills development, assessment support, or continuing professional development. Context: The context included all educational and professional learning environments, including universities, nursing and midwifery schools, hospitals, clinical practice settings, community healthcare settings, and online or blended learning environments worldwide. Studies were eligible if they met all PCC components and addressed podcasting within nursing or midwifery education or CPD. Quantitative, qualitative, mixed-methods, reviews, and relevant non-empirical publications were included if they provided information relevant to the review objectives. Studies published in English between January 2005 and December 2024 were eligible. Studies published in English between January 2005 and December 2024 were eligible. The review also incorporated non-empirical sources, such as narrative reviews and expert commentaries, which provided relevant insights, aligning with the scoping review’s aim to map the existing literature. Exclusions applied to non-educational or unrelated studies, opinion pieces (were operationally defined as editorials, viewpoints, commentaries, letters, or perspective articles that presented personal opinions without describing educational interventions, implementation experiences, or evidence relevant to the review objectives), lacking relevance (was defined as publications that did not address the use of podcasts for nursing or midwifery education or continuing professional development, even if they discussed podcasting in healthcare more broadly), or works not related to nursing or midwifery.

### 2.5. Search Strategy

The preliminary search of MEDLINE (via PubMed) and CINAHL was undertaken to identify relevant keywords, controlled vocabulary (Medical Subject Headings [MeSH] and CINAHL Headings), and indexing terms used in studies related to podcasting in nursing and midwifery education. The titles, abstracts, and indexing terms of relevant articles retrieved during this preliminary search were examined and used to refine the final search strategy applied across all databases. During the second search phase, both controlled vocabulary and free-text terms were used. Controlled vocabulary included database-specific indexing terms such as Medical Subject Headings (MeSH) in MEDLINE (e.g., Education, Nursing, Students, Nursing, Education, Continuing, Educational Technology) and equivalent CINAHL Headings where available. Free-text terms included. Podcast*, Nursing, Nurse*, Midwife*, Education, Continuing Professional Development, CPD, Lifelong learning, Digital learning.

These terms were combined using Boolean operators (AND/OR) and adapted to the indexing requirements of each database. The final search strategy was developed using terminology identified during the preliminary search. The search terms were adapted to reflect the differing indexing systems of MEDLINE and CINAHL, explicitly addressing the descriptors used in each database. Third, the reference lists of included studies were screened for additional eligible publications. A complete search strategy is available in the Supplementary Materials. The literature search was conducted independently by two reviewers (M.K., R.G., and YM), who executed the electronic database searches using the finalized search strategy. Duplicate records were identified and removed jointly by the three reviewers. Subsequently, JK, with the same reviewers, independently screened titles and abstracts against the eligibility criteria and performed full-text assessment of potentially eligible studies. Any disagreements regarding study eligibility were resolved through discussion, with arbitration by a third reviewer, AK, when consensus could not be reached.

### 2.6. Critical Appraisal of Evidence

This scoping review mapped and summarized the literature on podcasting in nursing, midwifery education, and CPD without formal bias assessment. Included studies varied in design, quantitative, qualitative, mixed-methods, and non-empirical—limiting firm conclusions. Findings suggest benefits such as flexibility, engagement, and knowledge retention, but the limited number and heterogeneity of studies preclude definitive claims of effectiveness.

### 2.7. Selection of Sources of Evidence

In the third stage of the review, titles and abstracts were independently screened by two reviewers against the predefined inclusion criteria. Full-text articles were subsequently assessed for eligibility. Discrepancies were resolved through discussion or consultation with a third reviewer to ensure consistency and methodological rigor. All search results were exported to reference management software (EndNote^TM^ 2025) and screened for duplicates. Two reviewers independently screened titles and abstracts against the inclusion criteria. Full texts of potentially relevant articles were then assessed. Any disagreements were resolved through discussion or consultation with a third reviewer. The selection process was documented using the PRISMA-ScR flow diagram [21].

### 2.8. Data Charting

Data charting represented the fourth stage of the scoping review. Relevant information was independently extracted by two reviewers using a standardized data extraction form developed in Microsoft Excel, covering study characteristics, educational context, podcast intervention details, and reported outcomes. Discrepancies between reviewers were resolved through discussion or, when necessary, with the involvement of a third reviewer to ensure consistency and accuracy. Data were independently extracted by two trained reviewers using a standardized data extraction form. The form captured key information, including author(s), year of publication, country, study design, population characteristics, podcast intervention details (format, frequency, delivery, and content), educational setting, reported outcomes, benefits, limitations, and any identified research gaps. Discrepancies between reviewers were resolved through discussion or, when necessary, with the involvement of a third reviewer to ensure consistency and accuracy. Data extraction also recorded whether studies explicitly reported a theoretical or pedagogical framework underpinning podcast design (e.g., adult learning theory, motivational frameworks), which was summarized descriptively rather than used as an inclusion criterion.

### 2.9. Data Analysis and Presentation

Data were analyzed using descriptive thematic analysis. Following data extraction, two reviewers independently examined the characteristics and findings of the included studies to identify recurring concepts related to the use of podcasting in nursing and midwifery education. An inductive approach was adopted to develop the thematic areas. Rather than applying predefined categories, themes emerged from repeated reading and comparison of the extracted data. Initially, meaningful codes representing key study findings were assigned to the extracted information. Similar codes were then grouped into broader categories based on conceptual similarity.

Through an iterative process of discussion and comparison, these categories were refined and consolidated into overarching thematic areas that reflected the aims of the review. During refinement, overlapping categories were merged, ambiguous themes were clarified, and category labels were revised to improve conceptual coherence and ensure that each theme accurately represented the included evidence.

To enhance analytical consistency and credibility, two reviewers independently coded the extracted data and compared their coding decisions. Discrepancies were resolved through discussion and consensus. When agreement could not be reached, a third reviewer adjudicated the final decision. The final thematic framework was subsequently used to organize the narrative synthesis and presentation of the review findings.

## 3. Results

### 3.1. Description of the Selected Studies

**Figure 1:**
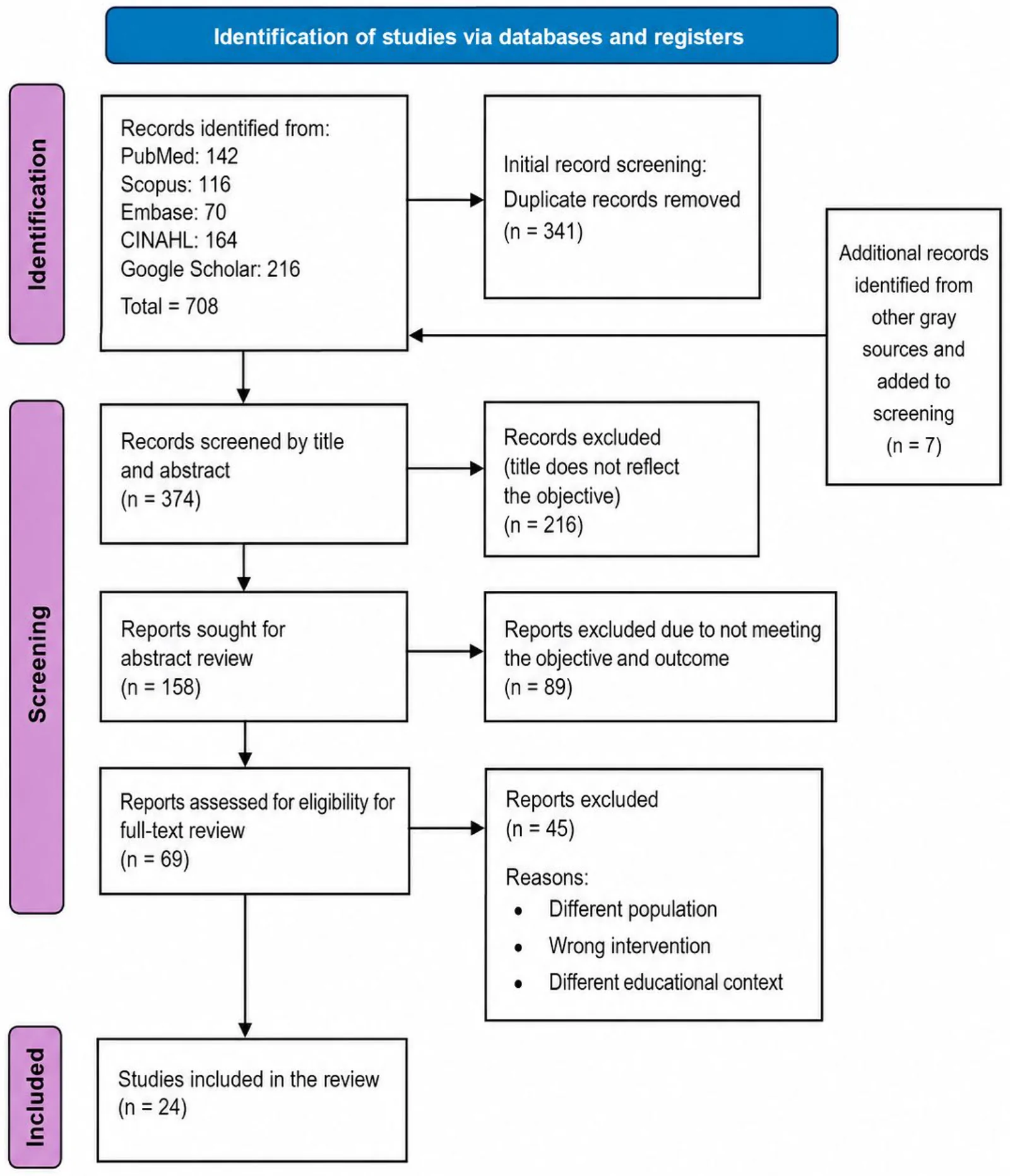
PRISMA-SCR flow diagram.

The reviewed literature includes 24 studies with diverse research designs (Figure 1). Quantitative approaches were most common (10 studies), featuring pre- and post-test designs, randomized controlled trials, and descriptive surveys. Five studies employed qualitative methods, including hermeneutic phenomenology and participatory design; another six used a mixed-methods approach that combined surveys, focus groups, or intervention assessments. Three papers were non-empirical: an integrative review, a systematic review, and a commentary exploring the use of podcasts in health education. This range highlights a growing, methodologically diverse interest in podcast-based interventions and education (Table A1). Although the review aimed to capture the literature on both nursing and midwifery education, most included studies focused on nursing populations. Only a small number of studies explicitly addressed midwifery education, reflecting both the limited availability of midwifery-specific podcasting research and the structural variability of midwifery education across countries, particularly in contexts such as the United States, where midwifery education is less widely represented within mainstream academic institutions.

### 3.2. Collating, Summarizing, and Synthesizing the Results

Stage 5 of the scoping review involved the systematic collation and synthesis of findings from the 24 included studies to map how podcasting has been utilized across nursing education, midwifery education, and continuing professional development (CPD). Rather than evaluating effectiveness, this stage focused on identifying patterns of use, dominant themes, and areas of convergence and divergence across educational contexts, in line with the purpose of scoping reviews. The synthesis yielded four interrelated thematic domains: educational function of podcasts, learner experience and engagement, pedagogical and professional outcomes, and implementation challenges and limitations.

### 3.3. Educational Function of Podcasts Across Learning Contexts

Across the 24 included studies, podcast-based educational interventions demonstrated positive educational outcomes in nursing and midwifery education. Knowledge acquisition and academic performance were the most frequently reported outcomes, with 11 studies reporting improvements in learners’ knowledge, awareness, understanding, or examination performance following podcast-based interventions [12,15,22,23,24,25,26]. Among these, Abedian et al. reported a significant improvement in knowledge scores among midwifery students receiving podcast-based education compared with traditional teaching (*p* = 0.004) [27]. At the same time, Anderson et al. demonstrated substantial gains in students’ understanding of the Sustainable Development Goals [24]. Similarly, Abate reported superior knowledge retention and application among students receiving segmented podcasts compared with traditional lectures. In pre-licensure nursing and midwifery education, podcasts were most commonly used to reinforce lecture content, support revision, and provide preparatory or follow-up learning resources for clinical skills and theoretical concepts. Typical topics included physical assessment, history-taking, intravenous procedures, pharmacology, delirium awareness, and broader professional concepts such as caring theory and the Sustainable Development Goals [22,23,24].

Podcast formats varied across studies but were most frequently delivered as short, segmented audio recordings, often 5 to 15 min long, hosted on institutional learning management systems or on publicly accessible platforms such as iTunes U and podcast hosting services [9]. Several studies have highlighted the pedagogical advantages of segmented podcasts, reporting improved knowledge retention and application when content is divided into focused episodes rather than extended recordings [25].

In CPD contexts, podcasts functioned as flexible, just-in-time learning resources, enabling practicing nurses and midwives to engage with professional education alongside clinical responsibilities. CPD-oriented podcasts frequently addressed practice-relevant topics such as patient safety, anesthesia fundamentals, leadership, professional role development, and emerging clinical guidelines [26]. In some cases, podcast participation was formally recognized for CPD credit, indicating emerging institutional acceptance of podcasting as a legitimate professional development modality [3].

### 3.4. Learner Experience and Engagement

Learner perceptions of podcasting were consistently positive across educational levels and settings. They were reported in nine studies. Students consistently perceived podcasts as valuable learning resources that enhanced engagement and motivation. Quantitative findings indicated high satisfaction, with 85% of students reporting podcasts were useful for examination preparation, and 83% finding podcasts beneficial for revision [28]. Students and professionals reported that podcasts are accessible, flexible, and learner-centered, allowing learners to engage with content at their own pace and in their own location [29]. Emerged as key themes in eight studies. Learners valued the ability to access educational materials anytime, repeatedly review complex concepts, and integrate learning into clinical practice and daily activities. These findings were consistently reported across both quantitative and qualitative studies, highlighting podcasts as convenient and learner-centered educational resources that support independent and lifelong learning. The ability to pause, replay, and revisit content was frequently cited as supporting comprehension and revision, particularly in preparation for assessments or clinical practice [28,30].

Qualitative findings further emphasized the role of podcasts in enhancing learner autonomy, confidence, and self-efficacy. Students described feeling more prepared for clinical placements and assessments when podcasts were used alongside face-to-face teaching [31,32]. In CPD and outreach-oriented contexts, podcasts were also used to support health education and professional knowledge translation beyond formal academic settings, with nursing faculty reporting enhanced engagement and accessibility when using podcasts to disseminate perinatal health information to community audiences [33]. Clinicians and learners valued the opportunity to integrate learning into routine activities such as commuting or administrative tasks, reducing time-related barriers to professional development [16].

Several studies have also highlighted a preference for conversational and narrative podcast styles, particularly those that incorporate storytelling, expert interviews, or reflective dialogue, which are perceived as more engaging than didactic formats [23,24]. Co-designed podcasts involving students, educators, clinicians, and, in some cases, patients were associated with increased relevance and learner ownership.

### 3.5. Pedagogical and Professional Outcomes

Reported outcomes across the included studies primarily related to knowledge acquisition, engagement, and perceived learning effectiveness. Podcast-based interventions contributed to improvements in critical thinking, clinical confidence, communication skills, health education competencies, and professional development among undergraduate students, graduate nurses, nurse preceptors, and nurse educators. Although Blum (2014) observed greater improvements in critical thinking among students receiving podcasts, the differences were not statistically significant [26]. Qualitative studies further suggested that podcasts enhanced learners’ confidence, self-efficacy, reflective practice, and preparedness for clinical learning. Quantitative studies demonstrated statistically significant improvements in knowledge scores among learners exposed to podcast-based interventions compared with traditional teaching methods or alternative digital modalities [27]. Evidence also suggested that podcasts could support the development of critical thinking skills, although findings were mixed and often limited by small sample sizes and short follow-up periods [26].

In qualitative and mixed-methods studies, participants reported improved understanding of complex or abstract topics, increased confidence in applying knowledge to practice, and enhanced motivation to engage with learning materials [34]. In CPD contexts, podcasts were perceived as supporting reflective practice and professional identity development, particularly when aligned with real-world clinical scenarios and experiential learning [26].

From an institutional perspective, podcasts were identified as scalable and cost-effective educational resources that could be reused across cohorts and settings, thereby supporting blended learning and flipped-classroom approaches [3]. However, objective evidence linking podcast use to long-term clinical performance or patient outcomes remained limited across studies.

### 3.6. Knowledge Gaps and Future Research Directions

Analysis of the studies included identified several important gaps in the current evidence on podcasting in nursing and midwifery education. First, many studies employed small convenience samples from single institutions, limiting the generalizability of findings. Second, considerable heterogeneity existed in podcast design, duration, content, delivery platforms, and outcome measures, making comparisons across studies difficult. Third, most studies focused on short-term improvements in knowledge or learner satisfaction, whereas few evaluated long-term knowledge retention, behavioral change, clinical competence, or patient-related outcomes. Fourth, evidence regarding podcast use in continuing professional development and among practicing nurses and midwives remains limited compared with undergraduate education. Finally, relatively few randomized controlled trials or longitudinal studies were identified, highlighting the need for more rigorous research to establish the effectiveness and sustainability of podcast-based educational interventions.

### 3.7. Implementation Challenges and Limitations

Despite the reported benefits, the synthesis identified several recurring challenges in podcasting for nursing and midwifery education. A primary concern was the variability in content quality and pedagogical rigor, particularly in the absence of standardized guidelines or peer-review mechanisms for educational podcasts [18]. Differences in production quality, instructional design, and alignment with learning outcomes were evident across studies.

Another commonly reported limitation was the passive nature of audio-only learning, which may not suit all learners or learning objectives. Some participants reported difficulty maintaining attention during longer episodes, while others preferred visual or interactive learning modalities [7]. Furthermore, assessment and feedback mechanisms were rarely integrated into podcast-based interventions, limiting the ability to evaluate learning outcomes beyond self-reported measures [2,6].

Geographical and contextual disparities were also apparent. Most studies originated from high-income countries, with limited representation from low- and middle-income settings, where access to technology, digital literacy, and institutional support may differ substantially [12]. This imbalance restricts the generalizability of findings and highlights the need for context-sensitive research. The inclusion of studies focused on nurse-led public health education, such as community-oriented perinatal podcasts, further highlights the conceptual overlap between CPD, professional knowledge translation, and client education, underscoring the need for clearer boundary definitions in future research [33].

## 4. Discussion

As this review used a scoping methodology, the findings reflect how outcomes were reported in the literature rather than demonstrating causal effectiveness. No formal quality appraisal was undertaken; results should therefore be interpreted as indicative of trends, applications, and research gaps rather than as definitive evidence of educational impact. This scoping review identified the available literature on podcasting in nursing and midwifery education and CPD. Consistent with the review aims and research questions, the findings reveal a growing, methodologically diverse body of evidence supporting the use of podcasting as an additional digital learning strategy in pre-licensure and post-licensure learning settings [7].

In the 24 included studies, podcasting was mostly used as a supplement to regular teaching, not as a substitute. Educational implementations were observed in the undergraduate nursing and midwifery courses, clinical skills training, evaluation responses, and student progression processes. The patterns of utilization in the literature were consistent, indicating that podcasts were the most used technology, used to supplement theoretical material, reinforce clinical preparedness, and support flexible learning for students and practicing professionals [2].

The synthesis resulted in four overarching areas of themes. First, podcasts played an evident role in educational reinforcement, allowing learners to relearn complex information, study for exams, and revise clinical concepts. The podcast format, in the form of short segments, was particularly associated with better knowledge retention and learner satisfaction. Second, learners’ engagement and experience were very positive [35]. The accessibility, portability, and self-pacing of podcasts were appreciated by students and professionals alike, enabling learning outside the institution and accommodating clinical or personal obligations [36].

Third, the pedagogical and professional outcomes identified in the review were primarily associated with knowledge acquisition, confidence building, and perceived learning effectiveness. A few quantitative studies reported statistically significant improvements in knowledge scores following podcast listening, and qualitative evidence indicated increased self-efficacy, autonomy, and readiness to practice [37]. Within the CPD environment, podcasts were also found to facilitate lifelong learning, reflective practice, and professional role development, with clinically relevant content delivered in a narrative or conversational style. Lastly, the implementation problem was repeatedly reported. These were content-quality variation, the absence of standardized instructional design frameworks, inadequate integration of assessment strategies, and inconsistent evaluation of learning outcomes [38]. Notably, most studies were from high-income countries, with minimal representation from low- and middle-income settings, thereby limiting the global generalizability of current evidence.

Compared with other audiovisual learning modalities, podcasting offers greater flexibility and accessibility by supporting self-paced, asynchronous learning. While video-based resources may be more suitable for teaching procedural skills requiring visual demonstration, podcasts are particularly valuable for reinforcing conceptual knowledge and reflective learning [39]. These findings are consistent with Cognitive Load Theory and Adult Learning Theory, which support flexible, learner-centered educational approaches that promote self-directed learning and engagement [40].

Overall, the findings indicate that podcasting plays a meaningful role in contemporary nursing and midwifery education ecosystems. The evidence aligns with adult learning theories that emphasize autonomy, flexibility, and contextual learning, and supports podcasting as a scalable digital modality that can enhance engagement across the educational continuum. However, the literature remains largely exploration, with limited focus on long-term outcomes, behavior change, or clinical impact.

**Figure 2:**
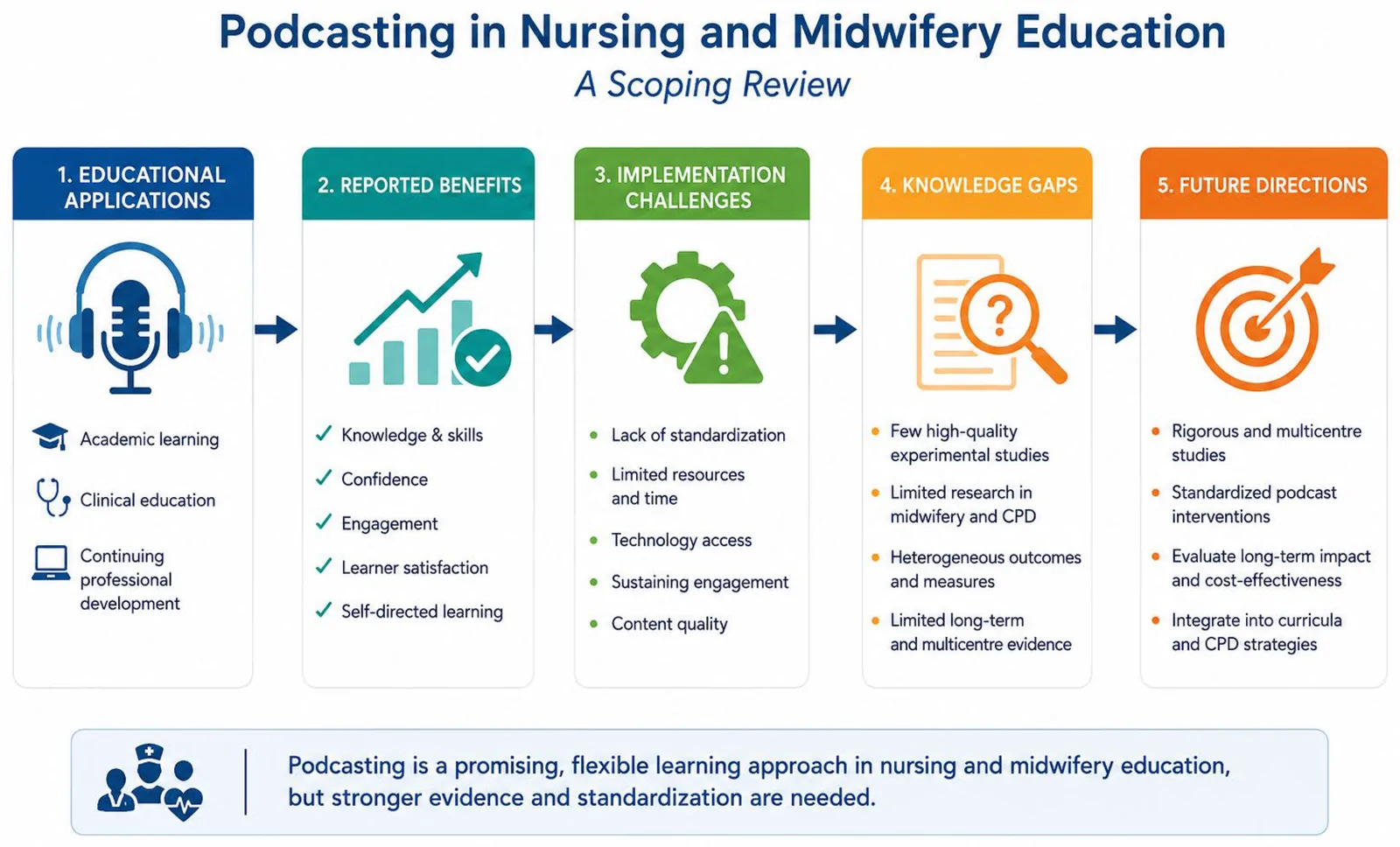
Conceptual figure—Podcasting in Nursing Education.

Although the included studies consistently reported positive learner perceptions and improvements in knowledge, confidence, and engagement, these findings should be interpreted with caution. As this review followed a scoping review methodology, no formal critical appraisal of methodological quality or risk of bias was conducted. Moreover, the included studies were heterogeneous with respect to study design, sample characteristics, podcast formats, educational settings, and outcome measures, limiting direct comparisons and precluding conclusions regarding effectiveness. Therefore, the findings should be viewed as mapping the current evidence rather than demonstrating the superiority of podcasting over other educational approaches. Similar educational benefits have also been reported in other health professions education disciplines, including medicine, pharmacy, and allied health, suggesting that podcasting represents a flexible digital learning strategy across healthcare education. Nevertheless, future research should employ robust experimental designs, standardized outcome measures, and long-term follow-up to establish its educational effectiveness and impact on clinical practice (Figure 2).

## 5. Limitations

The current scoping review has been conducted as a descriptive and thematic scoping review, rather than as a quality evaluation or the extraction of definitive conclusions. The inclusion of diverse paradigms, ranging from empirical studies to commentaries, resulted in heterogeneity that limits the generalizability of the findings and precludes statistical synthesis. Additionally, most studies relied on self-reported outcomes, which may have introduced bias.

An in-depth analysis of the current literature reveals several limitations. Podcast consumption is relatively under-supported by empirical evidence, with only a few quantitative studies on the topic. Thirdly, the literature presents an imbalance in research, as it tends to focus more on high-income settings, thereby underrepresenting low-income settings. Fourth, little is known about the topics or the form in which podcasts have the greatest impact, or about their optimal duration.

## 6. Recommendations

The integration of podcasts into nursing and midwifery education and professional development should be approached with careful planning, aligned with curriculum objectives, supported by high-quality instructional design, and backed by robust institutional infrastructure. Future research should prioritize rigorous quantitative studies, including randomized controlled trials, to evaluate the direct impact of podcasting on learning outcomes, clinical competencies, and patient care. It is also essential to explore the use of podcasts in low-resource settings and across diverse geographic regions to assess their potential to promote equitable access to education and advance global health outcomes. Additionally, investigations should examine the role of podcasts in interprofessional education and their influence on long-term behavior change among healthcare practitioners. Finally, the development and implementation of standardized tools to measure podcast engagement, learning effectiveness, and clinical impact are essential for generating robust, generalizable evidence to guide educational policy and practice.

## 7. Conclusions

This scoping review confirms that podcasting is increasingly adopted as an educational tool in nursing and midwifery education, as well as for CPD. It also offers significant benefits, including flexibility, accessibility, enhanced learner engagement, and improved knowledge retention. Educators and institutions can enhance podcast integration by aligning content with learning outcomes, incorporating it into blended learning models, and ensuring quality through structured design and evaluation. This scoping review mapped the current evidence on the use of podcasting in nursing and midwifery education. The available literature suggests that podcasting may serve as a flexible and accessible educational strategy that supports learner engagement, knowledge acquisition, and professional development across a range of educational settings. However, the evidence is heterogeneous with respect to study design, intervention characteristics, and outcome measures, and no formal quality appraisal was undertaken as part of this scoping review. Therefore, these findings should be interpreted as indicative rather than confirmatory. The review also identified important knowledge gaps, including the need for standardized podcast interventions, rigorous comparative studies, and long-term evaluations to better understand the educational impact of podcasting in nursing and midwifery education.

## Data Availability

No new data were created or analyzed in this study. Data sharing is not applicable to this article.

## Supplementary Materials

The following supporting information can be downloaded at: https://www.mdpi.com/article/10.3390/healthcare14182916/s1, File S1: Search Key words and Boolean Operators.

## Abbreviations

The following abbreviations are used in this manuscript:

CPD	Continuous professional development.
PRISMAscr	Preferred Reporting Items for Systematic Reviews and Meta-Analyses—Scoping Reviews.
MEDLINE	Medical Literature Analysis and Retrieval System Online.
CINAHL	Cumulative Index to Nursing and Allied Health Literature.

## Appendix A

**Table A1:** Data extraction table.

Author	Research Aims and Setting	Methods	Sample and Population	Intervention (Format, Frequency, Delivery, Content),	Results
(Abate, K.S., 2013) USA [25]	To evaluate the effectiveness of academic podcasts in promoting knowledge retention and application in nursing students. Setting: Undergraduate nursing education.	Design: Pilot study. Participants were randomized into 3 groups: traditional lecture, unsegmented podcast, and segmented podcast. Assessments: Multiple-choice quiz and case study.	35 female undergraduate nursing students.	Academic podcasts used in two formats: - Unsegmented podcast lecture - Segmented podcast lecture Compared to traditional face-to-face lecture.	Students in the segmented podcast group scored higher on knowledge retention and application measures than other groups. Implication: segmented format may enhance learning outcomes.
(Abedian, Z., 2018) Iran [27]	To examine the effects of podcasts on midwifery students’ knowledge regarding donor egg recipients. Setting: Academic institution.	Design: Pre- and post-test study. Data collection: Self-designed knowledge questionnaire and objective structured clinical exam.	Undergraduate midwifery students (n = 60, two groups of 30).	Developed by faculty. Content: Legal and religious issues related to egg donation. Duration: Three MP3 files (25 min each).	The podcast group showed a significant increase in knowledge scores (*p* = 0.004) compared to the traditional teaching group, though there was no significant difference in educational performance (*p* = 0.63).
(Anderson, T., 2024) UK [24]	To enhance nursing and midwifery students’ understanding of Sustainable Development Goals (SDGs) through a podcast. Setting: Queen’s University Belfast.	Design: Prospective study involving pre- and post-test questionnaires and focus group interviews.	566 first-year nursing and midwifery students.	A 60 min audio podcast co-designed with students and stakeholders, accessible via the university’s learning management system.	The podcast significantly improved students’ awareness and understanding of SDGs. Post-test scores indicated substantial gains in knowledge and perceived relevance. Participants found the podcast valuable, though some were uncertain about replaying it. Focus groups revealed themes of increased knowledge, behavioral changes, and a preference for the podcast format.
(Blum, C.A., 2014) USA [26]	To determine if podcast usage enhances students’ critical thinking abilities. Setting: Academic.	Design: Pilot interventional study with a pretest/post-test approach. Data collection: 34-item Health Sciences Reasoning Test (HSRT).	Nursing students in their final semester (control = 17; intervention = 21).	Developed by the researcher. Content: Supplemental podcast focused on critical thinking.	Students who engaged with the podcast exhibited a greater improvement in critical thinking skills compared to those receiving standard education (F(1, 36) = 1.91, *p* = 0.088). Overall HSRT scores did not reach statistical significance (F(1, 18) = 1.35, R^2^ = 0.037, *p* = 0.261).
(Kakhki, S.K., 2025) USA [17]	To examine the use of educational podcasts for nurse preceptors on their commitment to the preceptor role, perception of benefits, and perception of support. Setting: South Florida Hospital, USA	Design: Mixed-methodology correlation design with pre-experimental pretest/post-test	Staff nurses (n = 28) using a convenience sample	Surveys with three validated subscales, Podcast tracking data	Four educational audio and video podcast sessions were developed based on caring theory and the preceptor-identified hallmarks of unsafe practice (Attitude problems, poor communication skills, Inability to demonstrate knowledge and skills, and unprofessional behavior) through the Hospital’s internal network and YouTube channel
(Burke, S., 2014) USA [29]	To examine undergraduate nursing students’ perceptions of podcast materials, determine the benefits of podcasting, and determine when and where students used podcast materials. Setting: LaSalle University, USA	Design: Mixed Methodology Pretest/post-test pre-experimental design.	Undergraduate nursing students (n = 101) using a Convenience sample	Podcast perception survey, interviews	Podcast materials of traditional multiple lectures through the University learning management system (LMS) and on the iTunes U platform
(Coughlin, V., 2024) USA [15]	To compare the effectiveness of different educational modalities, podcasts vs. electronic newsletters, for clinical knowledge acquisition and self-efficacy among clinical nurses Setting: Adelphi University, USA	Design: A randomised control trial (RCT) Intervention group: Podcast Control group: Electronic newsletter	Clinical Professional Nurses (n = 113) using a simple random sample	Pretest/Posttest Knowledge Assessments Questionnaires	The podcast lectures about clinical knowledge were developed using sociocultural theory, which includes storytelling, Interactive dialogue, and Engagement cues based on cognitive and emotional learning principles through Podcast Hosting and Email
(O’Connor, S., 2020) Ireland [2]	To develop health information podcasts created by graduate nurses and midwives. Setting: University College Cork (UCC).	Design: Project involving podcast creation using recording studios or smartphone applications.	21 graduate nurses and midwives.	Each graduate developed a podcast on a health-related topic based on their expertise and experiences.	The project allowed students to communicate health information effectively, enhancing their clinical, academic, and interpersonal skills. Podcasts addressed health topics in an accessible format, promoting trust and engagement with the public.
(Crooks, S., 2023) Ireland [23]	To evaluate the effectiveness of a co-designed Parkinson’s Awareness audio podcast as an educational tool for improving knowledge, awareness, and practice among nursing students. Setting: Queen’s University Belfast, Northern Ireland	Design: Mixed-Methods pre-test/post-test design	Undergraduate Nursing Students (n = 332) using a Convenience sample	Pre-test/Posttest Knowledge Questionnaires and Focus Group discussion	A co-designed educational audio podcast (storytelling and expert interviews) at any convenient time within 30 days as an MP3 audio file within the Canvas Learning Management System (awareness of Parkinson’s disease (PD) through University Learning Management System (LMS) platform
(Denny, A., 2024) Multi country [12]	To evaluate the effectiveness of health-related podcasts in enhancing knowledge and awareness among nursing students.	Design: Pre- and post-podcast questionnaires assessing knowledge on gestational diabetes and mental health.	2395 undergraduate general nursing students from multiple universities: University College Cork (n = 850), University of Galway (n = 450), Mzuzu University in Malawi (n = 719), University of Fort Hare in South Africa (n = 376).	Health-related podcasts targeting gestational diabetes and mental health.	This feasibility study will assess the impact of podcasts across different income-level countries. Ethical approvals have been obtained, and data collection is ongoing until February 2024, with analysis expected in March 2024. Results show podcasts’ utility as educational tools in diverse settings.
(Egilsdottir, H.Ö., 2021) Norway [34]	To explore and elicit the perspectives of nursing students regarding digital learning resources as podcast supports for B-PAS learning and application in clinical rotation. Setting: Norway	Design: Qualitative Longitudinal participatory design approach.	Undergraduate Nursing Students (n = 20) using a Convenience sample	Focus group discussion, audio-recorded workshop	Three co-designed videos and podcast workshops about Physical assessment as vital signs and history-taking skills, notice of patient deterioration processes, and human bioscience through the University Learning Management System (LMS) platform
(Firmino, C.F., 2025) Portugal [6]	To evaluate the usability, acceptability, and suitability of podcasts as an educational tool in nursing education. Setting: School of Health, Lisbon and Tagus Valley Region.	Design: Exploratory, observational, and descriptive study using a self-completed questionnaire.	117 nursing students (ages 18–54) from a bachelor’s program.	Podcasts developed by faculty on vital signs, available on the institution’s Moodle platform.	Students rated podcasts as excellent tools for learning, with high usability scores (average 77.7). Podcasts enhanced motivation, flexibility, and control over learning, supporting self-directed education.
(Tiago, R.d.S., 2024) USA [1]	To evaluate the effectiveness of commercially prepared podcasts on enhancing critical thinking. Setting:	Design: Pilot study. Data collection: Online survey.	First-year nursing students (n = 20), with nine respondents.	Students selected one instructor-approved podcast episode for an assignment. Content: Nursing ethics.	All respondents (100%) felt the podcast assignment promoted learning; 66.6% would recommend incorporating podcast assignments in future courses.
(Kardong-Edgren, S., 2010) USA [30]	Aim: To evaluate the efficacy of lecture podcasts in supporting undergraduate nursing students’ learning and exam preparation. Setting: USA, undergraduate nursing education.	Design: Quantitative descriptive study. Data collected through surveys. Analysis used descriptive statistics.	210 undergraduate nursing students.	Lecture podcasts for Pathophysiology/Pharmacology courses. Delivered via iPod/MP3.	85% of students reported that podcasts were useful for exam preparation. High overall satisfaction rates with podcast use.
(McSwiggan, L.C. 2017) Scotland [7]	To explore students’ experiences with podcast feedback. Setting: Higher education institution in North-East Scotland	Design: Exploratory qualitative pilot study using focus groups. Analysis: Framework approach based on Self-Efficacy Theory.	Purposive sample of third-year undergraduate students.	Developed by faculty. Content: Podcasts aimed at enhancing students’ understanding.	Podcasts were found to enhance self-efficacy by providing accessible feedback and support for students.
(MacMullen, N., 2024) USA [33]	To explore the role of podcasts as a tool for health education, particularly for women of color regarding perinatal health.	Commentary discussing the use of podcasts alongside other social media platforms for health education.	Nursing faculty from Governors State University and community health representatives.	Educational podcasts focused on maternal health issues, particularly for women of color.	Podcasts enhance accessibility to health information, foster community engagement, and support educational outreach—positive feedback received from community members, highlighting the need for more health-related podcasts.
(Mitchell, G., 2021) Ireland [22]	To evaluate the effect of a delirium awareness podcast on undergraduate nursing student knowledge and confidence. Setting: Queen’s University Belfast in Northern Ireland.	Design: Quantitative Pre/Post-test study	Undergraduate nursing students (n = 320) using convince sample	Pre/post Knowledge Questionnaires	A delirium awareness lecture co-designed podcast workshop was developed for 60 min using free software from Audacity, an external microphone, and a laptop computer, and pre-and post-tests via a web link in the university’s Canvas LMS as an MP3.
(da Costa Mohallem, A.G., 2023) Brazil [9]	To evaluate the quality of podcasts produced by nursing lecturers as pre-class learning materials and assess lecturers’ engagement after a workshop. Setting: Nursing course at a higher education institution.	Design: Prospective, descriptive, and quantitative study. Data collection during the second semester of 2021.	23 nursing lecturers, with 18 participating (78%).	Lecturers produced 46 educational podcasts following a workshop on podcast creation.	Podcasts met quality criteria, with a mean access rate of 58% among students. All podcasts were informative, with an average duration of 6.2 min, aligning with expert recommendations. A significant percentage of lecturers successfully produced podcasts, indicating good engagement with the format.
(Mostyn, A., 2013) UK [28]	To explore nursing students’ perceptions of biology podcasts in a blended learning context. Setting: UK, multi-site undergraduate nursing programs.	Design: Mixed-methods design. Data collection through surveys and focus groups. Thematic analysis applied to qualitative data.	153 first-year undergraduate nursing students.	Supplementary biology podcasts provided as part of a blended learning approach. Accessed via WebCT.	83% of students found podcasts useful for revision. Reported improved understanding of content. Podcasts seen as effective supplementary learning materials.
(O’Brien, T., 2024) Multi country [4]	Aim: To explore the experiences of nursing students and nurses using podcasting and digital learning objects (DLOs) in nursing education. Setting: Systematic review of qualitative studies across various nursing education contexts.	Design: Qualitative systematic review using phenomenological synthesis. Thematic synthesis applied.	15 qualitative studies involving undergraduate nursing students and nurses.	Podcasts and other digital learning objects across various nursing education implementations. Content varied by study (e.g., traditional and digital lecture materials)	Podcasts were found to enhance learning due to flexibility, accessibility, and the ability to review content multiple times. Highlighted accessibility benefits and improved engagement with content.
(Merry, L., 2023) Canada [13]	To synthesize evidence on the use of podcasting in nursing and midwifery education. Setting: Various higher education institutions.	Design: Integrative review of literature. Databases searched: PubMed, MEDLINE, CINAHL, Scopus, ERIC.	Twenty-six studies included in the review.	Various studies utilized podcasting as a digital resource for nursing and midwifery education.	Podcasts contributed to new knowledge acquisition, improved clinical confidence, and facilitated flexible learning. Factors influencing effectiveness included digital literacy, organization, and content quality. The review calls for more robust research to assess the impact on learning outcomes.
(Rouhselang, S., 2020) USA [39]	To investigate educational podcasts as an adjunct to student registered nurse anesthetists (SRNAs) and measure their satisfaction. Setting: Marian University, USA.	Design: Mixed-methods translational research project using a post-intervention survey	Student Registered Nurse Anesthetists (SRNAs) (n = 26) using a Convenience sample	Podcasts Questionnaire (SSEPQ), Pre/Post Knowledge Tests	Educational podcast with 6 episodes based on the Keller ARCS Model focusing on foundational anesthesia content applicable to SRNAs. The podcast episodes included: “Clinical Flow: from OR set up through intubation,” “The Anesthesia Machine,” and “FaceTime Audio on Apple devices
(Stone, R., 2020.) Australia [31]	To explore the meaning of undergraduate nursing students’ experiences of using video podcasts as an adjunct to existing teaching methods in developing confidence in clinical skills. Setting: Metropolitan University, Australia.	Design: Qualitative (Hermeneutic phenomenology)	Second-year Undergraduate nursing students (n = 10) using a purposive sample	Semi-structured face-to-face interviews.	Instructional three short audio and video podcasts, face-to-face learning in an acute medical/surgical nursing practice course about clinical competencies (intravenous infusion set-up and delivery, intravenous antibiotic bolus delivery, and intramuscular injection) through University Learning Management System (LMS) platform
(Vogt, M., 2010) USA [40]	To examine the impact of podcasting on nursing student learning and satisfaction Setting: Otterbein College, Ohio, USA	Design: Quantitative comparative study	Undergraduate junior nursing students (n = 120) using a Convenience sample	Knowledge and satisfaction Surveys	Traditional face-to-face lectures, podcast lectures, voice-over PPT. Identical multiple-choice exam questions (two exam questions related to health promotion, two exam questions related to growth and development, and five exam questions related to childhood immunizations) through mp3 player via the University LMS platform
