# Supplementary material for: Podcasting in Nursing and Midwifery Education and Continuing Professional Development: A Scoping Review
Source: Healthcare (Basel). 2026 Sep 9;14(18):2916. doi: 10.3390/healthcare14182916 (PMC13606579; doi:10.3390/healthcare14182916)
Supplement: Supplementary file 1 [file healthcare-14-02916-s001.zip › healthcare-4329096-supplementary.pdf]

Search Key words and Boolean Operators.

PubMed:

("Podcast*"[Title/Abstract] OR podcasting [Title/Abstract]) AND ("Education, Nursing"[Mesh]

OR nurse*OR nursing OR nursing students OR midwi*OR midwife*)AND(education OR CPD

OR "continuing professional development")

Limits: English 2005–2024

Scopus:

TITLE-ABS-KEY

(podcast*OR podcasting) AND TITLE-ABS-KEY

(nurse*OR nursing OR "nursing student*"OR midwi* OR midwife*) AND TITLE-ABS-KEY

(education OR "continuing professional development "OR CPDOR "professional development" OR "lifelong learning") AND PUBYEAR > 2004 AND LANGUAGE(English).

Embase (Elsevier)

(podcast OR podcast*OR podcasting) AND ('nursing education'/exp OR 'nursing student OR nurse*OR nursing OR 'nursing student*'OR 'midwifery education'/exp

OR midwi*OR midwife*) AND(continuing education OR education OR 'continuing professional development OR CPD: OR 'professional development 'OR 'lifelong learning')AND [English] AND [2005-2024].

CINAHL (EBSCOhost)

((MH "Podcasts")OR TI podcast*OR AB podcast*OR TI podcasting OR AB podcasting)AND((MH "Education, Nursing")OR (MH "Students, Nursing")OR (MH "Nursing Education")OR TI nurse*OR AB nurse*OR TI nursing OR AB nursing OR TI "nursing student*"OR AB "nursing student*"OR TI midwi*OR AB midwi*OR TI midwife*OR AB midwife*)AND((MH "Continuing Education")OR TI education OR AB education OR TI "continuing professional development "OR AB "continuing professional development "OR TI CPDOR AB CPDOR TI "lifelong learning "OR AB "lifelong learning")Limiters:English Language Published: January 2005–December 2024

Google Scholar:

podcast OR podcasting

nursing OR midwifery

education OR "continuing professional development" OR CPD
